# Safe from Scarlet Fever

**Published:** 1887-09-24

**Authors:** Edwin Chadwick


### SAFE FROM SCARLET FEVER.

By Edwin Chadwick, C.B.

As no instructions have yet been issued that I have seen calculated to meet the prevalent epidemic of scarlatina, of which a further advance is apprehended, I find it due to state to you the experiences which appear to be available for defence against the disease. It cannot be too widely made known that the readiest and the most powerful factor of prevention is "wash and be clean" by frequent head-to-foot washing with tepid water. Health Officers who have served in the most rife plagues have declared that they owed their immunity to such ablutions twice a day.

A Trained Nurse's Experience.

I have the notes of the experiences of a trained hospital nurse of twenty years' practice, who acted as a specialist in the treatment of cases of scarlatina. She states that her chief practice was to isolate the patient in a single room, the upper room if possible, and let no one else enter it; to so arrange as to keep the door and part of the window open in order to let a current of air pass through the room over the patient; to observe all the details of regulations as to cleanliness of the patient and the articles of clothing and furniture. For her own personal protection, never to drink out of the same vessel that had been used by the patient, to wash from head to foot with tepid water, and to change her clothes once a day. With these precautions she had never had a single case of the spread of the disease to a member of the family or anyone else during the twenty years, nor had she once contracted the disease herself.

In Working-class Homes.

This, however, is the high and effective application of sanitary science, but with the poorer classes the patient is commonly kept in the vitiated atmosphere of the crowded cottage. The wife cannot understand or obey orders not to admit anyone. Gossips come in with their children, and the disease is spread about. Well-appointed fever-wards are the only places of comparative security for such cases; and when the patient is washed and put into a clean bed, with less impure air, he is commonly on his way to recovery before the physician can be brought to examine him.

School Hygiene.

In schools the experience of the effects of cleansing the place and the removal of cesspits and foul drains ere the cleansing was effected, reduced the death-rate in one orphan institution from twelve in the thousand to eight in a thousand; whilst the cleansing of the person by a constant ablution with tepid water caused a reduction to four in a thousand. Other experiences tend to establish the value of personal cleanliness as a preventive factor and a reduction of deaths to less than one-third.

District Half-time Schools.

In the district half-time schools, with air clear from putrid, stagnant sewage in and out of the premises; with clean clothes and clean bed-linen; with only one child sleeping in each bed; with equal warmth and good ventilation; and with much manual exercise in the open air—under these conditions the children's diseases are banished. At the district half-time school of Anerley, out of some nine hundred children they have had no measles for twelve years, nor any scarlatina of spontaneous origin, nor any whooping-cough, nor any typhoid for years, except one outbreak which followed the bursting of a bad drain. These institutions are children's hospitals, and at the new large half-time school of the parish of Lambeth, at Lower Norwood, to which I recently obtained the visit of H.I.H. the Crown Princess of Germany (when she was staying with his Imperial Highness at Upper Norwood), whilst the probationary wards were rife with the children's diseases, her Imperial Highness received the testimony that from within the school itself the children's diseases of spontaneous origin were absolutely banished. I have obtained the separate and independent experiences of a number of others of these large half-time institutions, which entirely confirm the power of the factors of sanitation, completely applied, to prevent those diseases of children and of adults which cause so large a proportion of the general death-rates of the population. At one Congress the representatives of the Boarding-out Association told us that the deaths of the children who can have the summer cottage appliances were 2 per cent., or twenty in a thousand!—which is six times heavier than in the district half-time schools.

Jet Baths for Schools and Workshops.

A large proportion of people of the poorer classes have not means to provide proper appliances for washing for their children or for themselves. For their children appliances ought to be provided at the schools. For soldiers in France a two-handled pump is provided; the soldier steps into a tray of tepid water, soaps himself, from head to foot, when a jet of tepid water out of a tank which has one of cold to two of boiling water is played upon him; he dries himself with a towel and then dresses —all in five minutes' time. As against this process the method in use here requires seventy or eighty gallons of water in the bath and a period of twenty minutes for its carrying out. The cost of the more speedy cleansing by jet is a tenth of a penny—soap included. Jet baths ought to be provided for adults in factories, as well as for children at schools, as means of economy of food as well as of health. Sir Henry Doulton, I may state, has provided the new cleansing apparatus, and it was displayed at the International Health Exhibition.

Personal Protection.

For their own personal protection every Health Officer, every sanitary inspector, and everyone else, may be recommended to sponge freely with tepid water, and to wipe himself clean with a dry towel once at least every day, and twice during the epidemic period.

School Inspectors.

The duties of the Officer of Health include weekly visits and examinations of children at the infant and the primary schools. In going over the school with him the schoolmistress would point out to the inspector, or he would observe, the child with premonitory symptoms to be looked at—the cold shivers, the pains in the head—and would separate it from the rest, go home with it, examine the state of the habitation, and give orders for the separation of the healthy children and the isolation of the sick, giving the requisite directions for its treatment. A trained nurse would follow with more frequent visits to see that the directions were carried out. The regulations should provide for similar visits and examinations of places of work; the separation of the workers, followed by visits to the habitation, and by the removal, as far as possible, of the injurious conditions found there.

A Word to the Wise.

If the wage-classes could be made aware of the great importance of the measures that are provided for their benefit, but are not brought into operation, they might, through their representatives, bring them to bear for their protection at an expense of less than one-third of the insurance charges to which they are subjected.

